# Special Issue “Adductomics: Elucidating the Environmental Causes of Disease”

**DOI:** 10.3390/ht8030017

**Published:** 2019-07-31

**Authors:** Sofia A. Pereira, Alexandra M. M. Antunes

**Affiliations:** 1 CEDOC, Chronic Diseases Research Centre, NOVA Medical School, Faculdade de Ciências Médicas, Universidade NOVA de Lisboa, 1169-006 Lisboa, Portugal; 2 Centro de Química Estrutural, Instituto Superior Técnico, ULisboa, 1049-001 Lisboa, Portugal

**Keywords:** protein adducts, DNA adducts, mercapturates, mass spectrometry, exposure-related diseases, reactive metabolites, human exposure

## Abstract

Adductomics studies represent effective tools for providing additional insights into how exposure to reactive metabolites can underlie disease mechanisms. This special issue is focused not only on summarizing the analytical methodologies used for DNA, protein, and mercapturic acid adductomics tools but also on highlighting the opportunities and challenges for the application of this type of studies in biomedical research.

Adductomics represents a unique “omic” approach in biomedical research that allows for the characterization and quantification of exposure to individual exogenous and endogenous reactive compounds. This exposure is influenced by genetic, environmental, and life-style factors and is characterized by high inter-person variability and a life-time dimension. In practice, adductomics studies consist of the identification and quantification of covalent adducts resulting from the irreversible covalent modification of biomacromolecules (nucleophilic groups of DNA, lipids, proteins, and peptides) by reactive compounds; which are primarily electrophiles, can be of exogenous origin or endogenously generated. Regardless of their origin, the biomonitoring of reactive metabolites is difficult due to their short-life in vivo. In contrast, the covalent adducts afforded are stable and can be identified and quantified by adductomics tools, thereby providing a surrogate measure of exposure to reactive metabolites, acting as biomarkers of exposure and/or effect. Actually, the formation of covalent protein and DNA adducts with reactive metabolites may per se elicit functional alterations, initiate mutagenicity/carcinogenicity or trigger an immune response.

Adductomics studies can be targeted, when focused on the identification of covalent adducts formed upon exposure to a specific chemical agent, or untargeted, aimed at comprehensively characterizing the totality of covalent conjugates bound to a nucleophile. Regardless of the type of adductomics studies, mass spectrometry (MS)-based methodologies, particularly high-resolution MS, currently constitute the most suitable analytical platform, providing not only quantitative information about the extent of the covalent conjugation but also the unbiased identification of the covalent conjugate as well as the adduction site within the bionucleophile.

This special issue highlights the potentialities of adductomics in elucidating the environmental causes of disease. With contributions from several world-renowned experts in the adductomics field, it is focused not only on summarizing the analytical methods used for adductomics studies but also on highlighting the opportunities and challenges for the application of adductomics tools in biomedical research. In fact, adductomics studies represent an opportunity for the identification of risk factors and molecular mechanisms of chemical exposure-related adverse health outcomes. Additionally, this “omic” approach may allow for the identification of suitable biomarkers for patient stratification in precision medicine strategies and for disease diagnosis/prognosis. Additionally, adductomics studies may provide evidence for guiding regulatory and preventive actions. Therefore, the investment in novel high-throughput methodologies for adductomics studies is needed and is anticipated to allow further expansion of this area with peculiarities that distinguish it from other “omics” strategies.

S.M. Rappaport and M. Törnqvist established themselves as pioneers in the field of untargeted protein adductomics by developing analytic platforms to characterize the totality of covalent adducts formed in the nucleophilic hot-spots, *N*-terminal valine and Cys 34, of the model proteins hemoglobin and human serum albumin, respectively. In this special issue, these renowned experts in adductomics, from the University of California and Stockholm University, joined efforts with Carlsson [1] to provide an overview of untargeted protein adductomics strategies, addressing critical aspects of this approach regarding the choice of target protein, sample preparation, analytical approach, and data analysis/identification of unknown adducts. 

R. Turkeys’ research (University of Minnesota) was pivotal for the development of untargeted DNA adductomics area. In this special issue Guo and Turesky [2] summarized the LC-MS strategies used for DNA untargeted adductomics studies that are devoted to the simultaneous screening of multiple DNA adducts formed with direct and indirect genotoxicants, with no prior knowledge of the adducts in the sample. Importantly, these authors emphasized the need for novel bioinformatics tools to advance DNA adductomics technology. The demand for novel paradigms in data-processing steps in protein adductomics studies was also shared by A. Antunes’s group [3]. This group from ULisboa, focused on the development/application of adductomics tools for disease diagnosis and prognosis also highlighted the need for the developing of new MS-based workflows focused on protein adductomics. Another Portuguese group from NOVA University pointed out that beyond DNA and protein adductomics, the individual mercapturomic profile might be a key pathophysiologic factor in the onset of non-communicable chronic diseases [4]. One additional contribution for this special issue, led by R.G. Solomon from Case Western Reserve University, represents an illustrative example of how adductomics studies involving endogenous reactive metabolites can provide further insights into molecular mechanisms underlying diseases [5].
